# Genomic Analysis of Endophytic *Bacillus*-Related Strains Isolated from the Medicinal Plant *Origanum vulgare* L. Revealed the Presence of Metabolic Pathways Involved in the Biosynthesis of Bioactive Compounds

**DOI:** 10.3390/microorganisms10050919

**Published:** 2022-04-27

**Authors:** Giulia Semenzato, Tania Alonso-Vásquez, Sara Del Duca, Alberto Vassallo, Christopher Riccardi, Marco Zaccaroni, Nadia Mucci, Anna Padula, Giovanni Emiliani, Antonio Palumbo Piccionello, Anna Maria Puglia, Renato Fani

**Affiliations:** 1 Department of Biology, University of Florence, Via Madonna del Piano 6, Sesto Fiorentino, 50019 Florence, Italy; giulia.semenzato@unifi.it (G.S.); tania.alonsovasquez@unifi.it (T.A.-V.); sara.delduca@unifi.it (S.D.D.); alberto.vassallo@unicam.it (A.V.); christopher.riccardi@unifi.it (C.R.); marco.zaccaroni@unifi.it (M.Z.); 2 Institute for Environmental Protection and Research, Via Ca’ Fornacetta 9, Ozzano dell’Emilia, 40064 Bologna, Italy; nadia.mucci@isprambiente.it (N.M.); anna.padula@isprambiente.it (A.P.); 3 Institute for Sustainable Plant Protection (IPSP), National Research Council (CNR), Via Madonna del Piano 10, Sesto Fiorentino, 50019 Florence, Italy; giovanni.emiliani@ipsp.cnr.it; 4 Department of Biological, Chemical and Pharmaceutical Sciences and Technologies-STEBICEF, University of Palermo, Viale delle Scienze Ed.17, 90128 Palermo, Italy; antonio.palumbopiccionello@unipa.it (A.P.P.); a.maria.puglia@unipa.it (A.M.P.)

**Keywords:** bacterial endophytes, essential oil, antimicrobial resistance, microbiome, plant growth-promoting bacteria

## Abstract

Multidrug-resistant pathogens represent a serious threat to human health. The inefficacy of traditional antibiotic drugs could be surmounted through the exploitation of natural bioactive compounds of which medicinal plants are a great reservoir. The finding that bacteria living inside plant tissues, (i.e., the endophytic bacterial microbiome) can influence the synthesis of the aforementioned compounds leads to the necessity of unraveling the mechanisms involved in the determination of this symbiotic relationship. Here, we report the genome sequence of four endophytic bacterial strains isolated from the medicinal plant *Origanum vulgare* L. and able to antagonize the growth of opportunistic pathogens of cystic fibrosis patients. The in silico analysis revealed the presence of gene clusters involved in the production of antimicrobial compounds, such as paeninodin, paenilarvins, polymyxin, and paenicidin A. Endophytes’ adaptation to the plant microenvironment was evaluated through the analysis of the presence of antibiotic resistance genes in the four genomes. The diesel fuel degrading potential was also tested. Strains grew in minimum media supplemented with diesel fuel, but no *n*-alkanes degradation genes were found in their genomes, suggesting that diesel fuel degradation might occur through other steps involving enzymes catalyzing the oxidation of aromatic compounds.

## 1. Introduction

The rapid development of multidrug-resistant pathogens requires novel therapeutic chemical compounds, of which medicinal and aromatic plants represent a great reservoir. The discovery that microorganisms residing inside the plant tissues may produce similar, if not the same, bioactive compounds as their plant hosts could offer a new sustainable and promising source of bioactive molecules [1,2].

Endophytes are referred to as microorganisms that reside inside the inner tissues of plants without any visible signs of infection [3]. They can influence plant physiology by facilitating nutrients acquisition, producing or modulating the levels of phytohormones, and inducing systemic resistance in their host [4]. The bacterial infection contemplates more phases regulated by the activation of gene expression in both plant and microorganisms: first, the production of root exudates attracts soil bacteria near the rhizosphere; once the bacteria have adhered to the surface of the roots, they can penetrate inside the internal parts of the plant and colonize different anatomical parts [5]. It has been demonstrated that different compartments of the same plant are inhabited by different endophytic communities: the cultivable bacteria obtained from the anatomical structures of the medicinal plants *Echinacea purpurea* L. and *Lavandula angustifolia* Mill. are characterized by a distinct structure and composition in terms of genera and endophytic strains [6,7]. The peculiar distribution of the endophytes within plant inner tissues suggests the existence of a selective pressure, but little is known about the forces involved in the shaping of the microbial communities.

The plant anatomical parts represent specific niches that require endophytes adaptation. Antimicrobial molecules produced by specialized compartments of medicinal plants could allow microorganisms with particular antimicrobial resistance characteristics to colonize that specific tissue; thus, essential oils produced by aromatic plants could act as a selective force driving microbial communities distribution inside the plant [8]. Moreover, bacterial endophytes themselves might influence the microenvironments of the plant [9]: the microorganisms can strongly influence the plant’s bioactive molecules production, contribute to the secondary metabolism of their host, and directly synthesize antimicrobials [10].

In this context, metaomics and comparative genome analysis can be applied to the plant holobiont to unravel the mechanisms implied in the plant–microbiota interactions. Identification and characterization of genes involved in such a beneficial relationship are necessary to better exploit the mutualistic association between the two counterparts [11]. Functional aspects and molecular interactions can be delineated, enabling the prediction of endophytes’ capacity to synthesize novel bioactive secondary metabolites and potential antibacterial drug candidates [4], able to overcome the global issue of antibiotic resistance.

In this work, we provided the genetic features of four endophytic strains belonging to a bacterial collection of isolates obtained from the medicinal plant *O. vulgare* L., widely known for its antimicrobial and antioxidant activity [12]. Based on the inhibiting activity exerted by some of the bacterial isolates tested against human pathogens [13], OVS6, OVL9, OVF10, and OVS21 were selected as good candidates for further characterization. Thus, their genomes sequence was obtained, in order to decipher the genetic features that underlie the plant–microbiota interactions and to better understand the potential pharmacological applications of endophytes.

## 2. Materials and Methods

### 2.1. Bacterial Strains and Growth Conditions

Plants of *Origanum vulgare* L., cultivated in the common garden “Giardino delle Erbe—Augusto Rinaldi Ceroni” (Casola Valsenio, Ravenna, Italy) were collected in July 2018. Endophytes and soil bacteria were isolated from the anatomical parts of the plant, (i.e., flower, leaf, and stem) and bulk soil, as described in Castronovo et al. [13]. One isolate from flower, one from leaf, and two from stem compartments were selected for the present work. Isolates are referred to as OV followed by F, L, or S for flower, leaf, and stem districts, respectively. Three of them were previously affiliated with the *Bacillus* genus (OVL9, OVS6, and OVS21), while OVF10 belongs to the genus *Paenibacillus* [13]. Each strain was cultured on tryptic soy agar (TSA) medium (BioLife Italiana, Milan, Italy) for 48 h at 30 °C.

### 2.2. Tests for the Diesel Degrading Potential of the Endophytes

The four endophytic strains were tested for their ability to grow in the presence of diesel fuel as the only carbon and energy source. A single colony of each endophyte was separately suspended in 100 μL of 0.9% *w*/*v* NaCl solution and then streaked on different minimum media agar plates supplemented with glucose or diesel. In particular, bacterial strains were streaked on Minimum Davis (MMD, 1 g (NH_4_)_2_SO_4_, 7 g K_2_HPO_4_, 2 g KH_2_PO_4_, 0.5 g Na_3_-citrate·2H_2_O, 0.1 g MgSO_4_·7H_2_O, pH 7.2, per liter of deionized water) and M9 minimal medium (6 g Na_2_HPO_4_, 3 g KH_2_PO_4_, 0.5 g NaCl, 1 g NH_4_Cl, 1 mL MgSO_4_ 1M, 0.5 mL CaCl_2_ 0.5M per liter of deionized water) containing 0.4% *v*/*v* diesel fuel or 1% *w*/*v* glucose as the sole carbon sources. Diesel fuel (Esso Italiana, Roma, Italy) was previously filtered through a 0.2 μm-pore-size filter (Sartorius) for sterilization and particle removal. TSA plates were used as growth control. Once streaked, the endophytes were incubated at 30 °C for 3 days. Positivity to this assay was assessed as the presence or the absence of visible growth, expressed in a range from 4 (complete growth) to 0 (absence of growth).

The test was then performed in liquid medium, using MMD and M9 supplemented with glucose (1% *w*/*v*) or diesel (0.4% *v*/*v*), and tryptic soy broth (TSB, BioLife) as growth control. Endophytes growth curves were performed in microplates. Cells were grown overnight at 30 °C under shaking (130 rpm) in TSB. The next morning, cell cultures were washed twice in saline solution (NaCl 0.9% *w*/*v*) and diluted to a starting O.D._600_ of 0.01 in each media. Cells were then incubated at 30 °C and the O.D._600_ measures were taken every hour for 72 h using the Infinite M Nano (Tecan, Männedorf, Switzerland) microplate reader. Each curve was performed in triplicate. The calculation of the areas under the growth curve (AUC) was obtained according to the chained trapezoidal rule [14]. Statistical significance of the differences between each condition was calculated through a non-parametric test and a post hoc analysis, Kruskal-Wallis and Dunn tests.

### 2.3. DNA Extraction

A single colony of each strain was inoculated in 10 mL of fresh TSB (Biolife) in a 50 mL tube and incubated at 30 °C overnight under shaking at 130 rpm. Bacterial cells were collected by centrifugation (15,500× *g* for 4 min) and the genomic DNA was extracted using PowerLyzer PowerSoil DNA Isolation Kit (MO BIO Laboratories, Inc., Carlsbad, CA, USA), following the protocol provided by the manufacturer with some modifications to ensure optimal cell lysis. In particular, cells were resuspended in the PowerSoil Bead Solution in the presence of lysozyme (final concentration 1 mg/mL); after incubating for 1 h at 37 °C, PowerSoil Solution C1 and proteinase K (final concentration 0.5 mg/mL) were added and samples were furtherly incubated at 55 °C for 2 h before proceeding with the DNA purification steps.

### 2.4. Genome Sequencing

Nanopore sequencing was performed with a PCR-free approach following the native barcoding genomic DNA protocol provided by Oxford Nanopore Technologies (ONT) (version NBE_9065_v109_revY_14Aug2019). The gDNA of the strains were sequenced with other 8 non-related gDNA samples. Briefly, 1 µg of each input gDNA was repaired and end-prepped using the NEBNext Companion Module for Oxford Nanopore Technologies Ligation Sequencing (E7180S, New England Biolabs). Upon purification with Agencourt AMPure XP beads (Beckman Coulter) on a magnetic separator, concentrations of DNA samples were determined using a Qubit 4 Fluorometer and Qubit dsDNA HS Assay Kit (ThermoFisher Scientific). Thus, 500 ng of each end-prepped DNA sample were barcoded using Native Barcoding Expansion 13–24 (EXP-NBD114, ONT) and NEB Blunt/TA Ligase Master Mix (M0367, New England Biolabs). After a purification step, equimolar amounts of barcoded DNA samples were pooled to have a total of 700 ng and were subjected to the adapter ligation. During the subsequent clean-up step, the DNA library was enriched with >3 kb-long fragments using the Long Fragment Buffer included in the Ligation Sequencing Kit (SQK-LSK109, ONT). DNA library was immediately sequenced; therefore, an R9.4.1 Flow Cell (FLO-MIN106D, ONT) was primed with a Flow Cell Priming Kit (EXP-FLP002, ONT). The library was loaded following the instruction provided by the protocol and sequencing was performed with a MinION MK1B (ONT) and the MinKNOW software v.21.10.4 for 72 h. Basecalling and demultiplexing were performed using Guppy v.4.3.4. 

### 2.5. Genome Assembly and Bioinformatic Analysis

*De novo* assembly was accomplished using Canu assembler software v.2.1.1 [15] and the quality of contigs was evaluated by QUAST v.5.0.2 [16]. These procedures were performed in a Galaxy environment (https://usegalaxy.eu, accessed on 3 September 2021). The assembled genome sequences were annotated using the NCBI Prokaryotic Genome Annotation Pipeline (PGAP) v.6.0 (https://www.ncbi.nlm.nih.gov/genome/annotation_prok/, accessed on 22 March 2022). The Average Nucleotide Identity (ANI) analysis was performed using FastANI v.1.3, with default options [17]. The newly assembled genomes were uploaded to ContEst16S to assess the presence of contaminants. This service also generates a maximum likelihood phylogenetic tree using 16S rRNA sequences. Using this information, the genomic sequences of the genera that are more closely related to the bacterial strains under investigation were downloaded. These were retrieved from the NCBI and used as reference input for the ANI analysis. The affiliated species were confirmed with the Basic Local Alignment Search Tool (BLAST) by searching 3000 bp long random regions of each query genome.

### 2.6. Secondary Metabolites Gene Clusters Detection

The antiSMASH v.6.0.1 webserver was used for the identification of gene clusters involved in the biosynthesis of secondary metabolites in genomes of bacteria [18]. Each query genome (OVS6, OVL9, OVR10, and OVS21) was uploaded as a FASTA format, and the analysis was performed using a strict method of detection in order to identify only well-defined clusters containing genes with a significant alignment.

### 2.7. Antibiotic Resistance and Diesel Fuel Degradation Genes

The Resistance Gene Identifier from the Comprehensive Antibiotic Resistance Database (CARD: RGI) was used to predict resistome(s) from genome data based on homology and SNP models [19].

Alkane hydroxylases gene sequences, *alkM*, *alkS*, and *alkT*, were downloaded from the NCBI gene database, and then compared with OVS6, OVL9, OVF10, and OVS21 genomes. To verify the result, all three gene sequences were looked for in each one of the genome sequences affiliated to the query genomes (*Metabacillus dongyingensis*, *Paenibacillus xylanexedens*, and *Priestia megaterium*) and *Bacillus* sp. using the Basic Local Alignment Search Tool (BLAST).

## 3. Results

### 3.1. Genome Sequencing and Assembly

The results of genome assembly of the sequencing reads performed through Canu assembler plugin is shown in Table 1. The annotation of the genomic sequences was performed using the NCBI GenBank annotation pipeline. The complete genome sequences are available in GenBank under the accession numbers CP092335 (OVS6), JALHBO000000000 (OVL9), CP094668 (OVF10), JALHBP000000000 (OVS21).

Given the previous affiliation of OVS6, OVL9, and OVS21 to the genus *Bacillus* [13], their ANI analysis was performed using *Bacillus* sp. genomes downloaded from the NCBI assembly database (http://www.ncbi.nlm.nih.gov/assembly/, accessed on 7 March 2022) as references but showed low ANI values (<90%). In the light of this observation, the closest clades of the maximum likelihood phylogenetic trees obtained with ContEst16S were used as reference genomes. This resulted in higher ANI values and the affiliated species were confirmed with BLAST. Using 7 *Metabacillus* genomes for OVS6, 33 *Priestia* genomes for OVL9 and OVS21, and 116 *Paenibacillus* genomes for OVF10, the ANI analysis revealed that the endophytes are bound to *Metabacillus dongyingensis* with a 99.61 ANI value, *Priestia megaterium* with a 99.46 and 99.25 ANI values, and with *Paenibacillus xylanexedens* with a 95.25 ANI value, respectively (Table 2).

### 3.2. Tests for The Diesel Fuel Degrading Potential of the Endophytes

Diesel fuel degrading potential of the endophytes was investigated, as described in Materials and Methods. As shown in Table 3, all the endophytes grew on MMD and M9 minimal agar media supplemented with glucose 1% *w*/*v* and, to a lesser extent, on the same media containing diesel fuel 0.4% *v*/*v*.

The test was also performed using liquid media, in triplicate. For each condition, endophytes growth curves were obtained, and the average AUC was calculated. Since data did not present a normal distribution and homoscedasticity, non-parametric tests (Kruskal–Wallis test and Dunn test) were used to reveal statistically significant differences between each growth condition.

**Figure 1:**
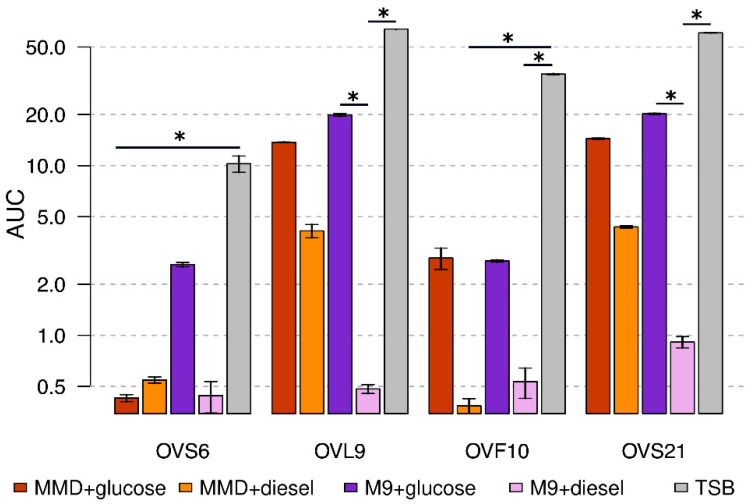
Diesel fuel degrading potential of the endophytes on liquid media. Values are expressed as the average values of area under the growth curve (AUC). Each color corresponds to a different growth medium, whereas bars represent standard errors. Asterisks show statistically significant differences between conditions (*p* < 0.05).

As shown in Figure 1, the two different minimal media supplemented with glucose allowed the growth of all the endophytes, even though to a lesser extent than the TSB complete medium, except for OVS6 which had an impaired growth in MMD with glucose. Statistically significant differences between different growth conditions are highlighted in Figure 1. Endophytes growth in MMD supplemented with diesel fuel was not statistically different from the MMD with glucose condition, thus allowing us to hypothesize the ability of the strains to grow in the presence of diesel fuel as a carbon and energy source.

To validate the results obtained, alkane-degradation genes were searched in the genomes of the endophytes. There was no presence of *alkM, alkS,* and *alkT* genes in all four query genomes. Their absence was confirmed using the Basic Local Alignment Search Tool and, as reference genomes, those of *Metabacillus dongyingensis*, *Paenibacillus xylanexedens, Priestia megaterium,* and *Bacillus* sp.

### 3.3. Secondary Metabolites Gene Clusters

The analysis of secondary metabolites biosynthetic gene clusters (BGCs) predicted several types of BGCs for each genome; however, only the ones with a percentage equal to or higher than 50% of similarity to clusters available in the database are reported (Table 4). For the OVS6 genome, the analysis revealed a lasso peptide BCG, accountable for the production of paeninodin; a 100% similarity was found to the paeninodin BCG from *Paenibacillus dendritiformis* C454. For OVL9 and OVS21 genomes, a terpene BCG was found, with a 50% similarity to the carotenoid BCG from *Halobacillus halophilus* DSM 2266. Finally, the analysis of the OVF10 genome showed three BCGs. The first two were attributed to the non-ribosomal peptide synthetase cluster, finding a 100% similarity to the paenilarvins BCG from *Paenibacillus larvae subsp. larvae* DSM 25430, and to the polymyxin BCG from *Paenibacillus polymyxa*. The third cluster was a lanthipeptide-class-I BCG, with an 85% similarity with the paenicidin A BCG from *Paenibacillus polymyxa*.

### 3.4. Antibiotic Resistance Genes

Using the Resistance Gene Identifier from the Comprehensive Antibiotic Resistance Database (CARD: RGI), for the OVF10 genome, one antibiotic resistance gene was found with a strict criterion algorithm. It obtained an identity of the matching region of 84.32% and 100% of the length of the reference sequence. This gene belongs to the “Llm 23S ribosomal RNA methyltransferase” antibiotic resistance orthology (ARO) and antimicrobial resistance (AMR) gene family and it is linked to the lincosamide antibiotic class. The reference genome to which the query gene matched has—as a resistance mechanism—the antibiotic target alteration.

Using the loose criterion for the other three query genomes, several genes were identified; however, only the ones with a high percentage of identity of matching region and length of reference sequence are reported here. For the OVS6 genome, the RGI found two matches. The first one obtained an identity of the matching region of 80.74% and 100.34% of the length of the reference sequence. It belongs to the “*vgbB*” ARO and “streptogramin *vgb* lyase” AMR gene family and it is linked to the streptogramin antibiotic class. It uses antibiotic inactivation as a resistance mechanism. The second one obtained an identity of the matching region of 80.6% and 99.57% of the length of the reference sequence. It belongs to the “*vanRM*” ARO and “glycopeptide resistance gene cluster, *vanR*” AMR gene family and it is related to the glycopeptide antibiotic class. It contemplates target alteration as a resistance mechanism. Lastly, the same hit was found for OVL9 and OVS21 genomes, which had an identity of the matching region of 80.45% and 80.53%, respectively, and 100.59% of the length of the reference sequence for both genomes. The hit gene belongs to the “*Staphylococcus aureus rpoB* mutants conferring resistance to rifampicin” ARO and to the “rifamycin-resistant beta-subunit of RNA polymerase (*rpoB*)” AMR gene family. It uses antibiotic target alteration and antibiotic target replacement as resistance mechanisms, and it belongs to the rifamycin antibiotic class. Since the loose algorithm works outside the detection model cut-offs, the found genes may require further study.

## 4. Discussion

Endophytes, microorganisms that reside in the internal tissues of living plants, have been recognized as potential untapped sources of novel natural products that could find applications in medicine, agriculture, and industry [20]. Endophytes are known to be able to synthesize some similar, if not the same, chemical compounds like the ones found in medicinal plant extracts, probably as an adaptation to the plant microenvironment [21]. Medicinal and aromatic plants have always been exploited for their natural biologically active compounds, so they can represent the starting point to investigate endophytes’ secondary metabolites biosynthesis potential [2].

The aim of this work was to determine and analyze the complete genome sequence of four endophytic bacterial strains isolated from stem or leaves of *O. vulgare* plants in order to identify genes involved in the biosynthesis of antimicrobial compounds and/or responsible for resistance to antibiotics.

The genome analysis of the four *O. vulgare* endophytes revealed some relevant genetic features providing insights into their chemical potential as antibiotics producers and helping to identify genetic traits that could be associated with the endophytic lifestyle. 

The ANI analysis allowed to shed light on the taxonomical position of the isolates, previously identified as *Bacillus* spp. (OVS6, OVL9, and OVS21) and *Paenibacillus* sp. (OVF10). The four *O. vulgare* endophytes belong to species already isolated from the aerial parts of other plants or their rhizosphere. Based on the ANI analysis results, OVS6 was identified as *Metabacillus dongyingensis*. This microorganism has been identified as a novel plant growth-promoting bacterium isolated from the saline-alkaline rhizosphere of *Ulmus pumila* and it is able to promote the growth of *Zea mais* L. under saline conditions [22]. Genome sequences from OVL9 and OVS21 showed a high degree of similarity to the species *Priestia megaterium*, formerly known as *Bacillus megaterium* [23]. *B. megaterium* isolated from the rhizosphere of maize plants demonstrated a growth-promoting effect on *Arabidopsis thaliana* that involved auxin and ethylene production [24]. Finally, OVF10 was identified as *Paenibacillus xylanexedens*. Species of the genus *Paenibacillus* are well-known plant endophytes able to exert a growth-promoting activity on their host and protect it from phytopathogens through the biosynthesis of antibacterial compounds [25]. Thus, it can be suggested that these *O. vulgare* endophytes could be plant growth-promoting bacteria, exerting a beneficial effect on their host.

Diesel fuel degrading potential tests revealed the capability of the four endophytic strains to grow in the presence of diesel fuel as the sole carbon and energy source. The essential oil composition of *O. vulgare* plant from which the endophytes were extracted revealed the presence of 73.5% sesquiterpene hydrocarbons, 17.6% monoterpene hydrocarbons, 4.8% oxygenated sesquiterpenes, and 3.7% oxygenated monoterpenes [13]. The capability of the endophytes to grow on media containing diesel fuel could suggest their adaptation to the hydrocarbon components of *O. vulgare* essential oil, leading to the hypothesis that its composition might represent one of the factors involved in the plant-endophytes symbiosis [8]. For this reason, alkane hydroxylases genes (*alk* genes) have been searched in the endophytes’ genomes. Even though these genes have not been identified, it cannot be excluded that the strains’ capability to grow on diesel fuel might depend on other gene clusters involved in different hydrocarbons metabolism.

Resistance Gene Identifier provided information about the antibiotic resistance profiles of the four isolates. Data obtained can be related to the adaptation mechanisms that endophytes require in order to colonize plant inner tissues, which are microenvironments rich in antimicrobial molecules.

The evaluation of secondary metabolites biosynthetic gene clusters revealed a wide spectrum of compounds. Concerning the OVS6 genome, the analysis revealed a lasso peptide biosynthetic gene cluster (BGC) accountable for the production of paeninodin; a 100% similarity was found with the paeninodin BGC from *Paenibacillus dendritiformis* C454. Lasso peptides are a class of ribosomally-synthetized and post-translationally modified peptides (RiPPs), known for their antimicrobial and antiviral activities [26]. OVF10 genome contains two non-ribosomal peptide synthase clusters, finding a 100% similarity with the paenilarvins BGC from *Paenibacillus larvae subsp. larvae* DSM 25430, and with the polymyxin BGC from *Paenibacillus polymyxa*. It also harbors a lanthipeptide-class-I BCG, with an 85% similarity with the paenicidin A BGC from *Paenibacillus polymyxa*. Paenilarvins, members of the iturins family, are characterized by a strong antifungal activity, able to contrast the growth of human and plant and pathogenic fungi [27]. Polymyxins are well-known antibacterial molecules with a narrow antibacterial spectrum, mainly against common Gram-negative bacteria. They are active against most members of the Enterobacteriaceae family, including *Escherichia coli*, *Enterobacter* spp., *Klebsiella* spp., *Citrobacter* spp., *Salmonella* spp., and *Shigella* spp. [28]. On the contrary, paenicidin belongs to the antibiotic class of lantibiotics, which are active against Gram-positive bacteria [29]. The identified BCGs might be involved in the growth inhibitory activity that the four endophytes exert against ten selected strains belonging to the *Burkholderia cepacia* complex, opportunistic pathogens able to infect immunocompromised cystic fibrosis patients, and other Gram-positive and Gram-negative human pathogens [13].

These data represent a further step in the comprehension of the role of the bacterial endophytes in medicinal plant secondary metabolism and might shed light on their biotechnological application in pharmaceutical and agricultural fields. To test whether bacterial endophytes are involved in the promotion of *O. vulgare* plant growth and/or influence the biosynthesis of secondary metabolites that can then flow in the plant essential oil/extract, an in vitro model for *O. vulgare* plants can be set up. In this way axenic *O. vulgare* plants might be inoculated with bacterial endophytic strains isolated from the same plants, thus allowing the comparison of the behavior of inoculated plants vs. control, (i.e., non-inoculated ones) from different viewpoints: growth, production of secondary metabolite, volatile organic compounds. The setup of this in vitro model is in progress.

## Figures and Tables

**Table 1:** General features of the genomes of the four endophytic strains.

	OVS6	OVL9	OVF10	OVS21
Assembly size	5,073,147	6,280,800	7,243,271	5,954,252
Number of contigs	1	12	1	11
GC%	40.09	37.54	45.89	37.68
Total genes	5510	7242	7168	6717
CDSs	5388	7053	7026	6496
rRNA	33	43	36	51
tRNA	84	138	102	162
ncRNA	5	8	4	8
Accession number	CP092335	JALHBO000000000	CP094668	JALHBP000000000

**Table 2:** Identity values for each endophytic strain.

	OVS6	OVL9	OVF10	OVS21
Species	*Metabacillus dongyingensis*	*Priestia megaterium*	*Paenibacillus xylanexedens*	*Priestia megaterium*
ANI value	99.61	99.46	95.25	99.25
BLAST ID%	100	99.89	92.81	99.57
BLAST query coverage%	100	100	99	100

**Table 3:** Diesel fuel degrading potential of the endophytes on agar media. The values and the colors indicate different growth levels of the strains compared to the growth control, that is: complete (4), strong (3), intermediate (2), weak (1), absent (0).

	OVS6	OVL9	OVF10	OVS21
MMD + glucose	1	2	1	2
MMD + diesel fuel	1	1	1	1
M9 + glucose	2	3	1	3
M9 + diesel fuel	1	1	1	1
TSA	4	4	4	4

**Table 4:** Biosinthetic gene clusters identified using AntiSMASH and having the higher percentage of similarity. Nucleotide positions within the genome are reported.

Genome	Type	From (nt)	To (nt)	Most Similar Known Cluster (% Similarity)
OVS6	Lassopeptide	2840151	2864059	Paeninodin (100%)
OVL9	Terpene (contig 11)	4862360	4883208	Carotenoid (50%)
OVF10	Non-ribosomal peptide synthetase cluster	4914549	4966941	Paenilarvins (100%)
		6908170	6988910	Polymyxin (100%)
	Class I Lanthipeptide	270456	296759	Paenicidin A (85%)
OVS21	Terpene (contig 1)	4569666	4590514	Carotenoid (50%)

## Data Availability

Not applicable.
